# Microbial responses to changes in flow status in temporary headwater streams: a cross-system comparison

**DOI:** 10.3389/fmicb.2015.00522

**Published:** 2015-06-04

**Authors:** Catherine M. Febria, Jacob D. Hosen, Byron C. Crump, Margaret A. Palmer, D. Dudley Williams

**Affiliations:** ^1^Department of Entomology, University of MarylandCollege Park, MD, USA; ^2^Chesapeake Biological Laboratory, University of Maryland Center for Environmental ScienceSolomons, MD, USA; ^3^School of Biological Sciences, University of CanterburyChristchurch, New Zealand; ^4^College of Earth, Ocean, and Atmospheric Sciences, Oregon State UniversityCorvallis, OR, USA; ^5^National Socio-Environmental Synthesis Center, University of MarylandCollege Park, MD, USA; ^6^Department of Biological Sciences, University of Toronto ScarboroughScarborough, ON, Canada

**Keywords:** bacterial diversity, microbial ecology, temporary streams, operational taxonomic unit (OTU)

## Abstract

Microbial communities are responsible for the bulk of biogeochemical processing in temporary headwater streams, yet there is still relatively little known about how community structure and function respond to periodic drying. Moreover, the ability to sample temporary habitats can be a logistical challenge due to the limited capability to measure and predict the timing, intensity and frequency of wet-dry events. Unsurprisingly, published datasets on microbial community structure and function are limited in scope and temporal resolution and vary widely in the molecular methods applied. We compared environmental and microbial community datasets for permanent and temporary tributaries of two different North American headwater stream systems: Speed River (Ontario, Canada) and Parkers Creek (Maryland, USA). We explored whether taxonomic diversity and community composition were altered as a result of flow permanence and compared community composition amongst streams using different 16S microbial community methods (i.e., T-RFLP and Illumina MiSeq). Contrary to our hypotheses, and irrespective of method, community composition did not respond strongly to drying. In both systems, community composition was related to site rather than drying condition. Additional network analysis on the Parkers Creek dataset indicated a shift in the central microbial relationships between temporary and permanent streams. In the permanent stream at Parkers Creek, associations of methanotrophic taxa were most dominant, whereas associations with taxa from the order Nitrospirales were more dominant in the temporary stream, particularly during dry conditions. We compared these results with existing published studies from around the world and found a wide range in community responses to drying. We conclude by proposing three hypotheses that may address contradictory results and, when tested across systems, may expand understanding of the responses of microbial communities in temporary streams to natural and human-induced fluctuations in flow-status and permanence.

## Introduction

Temporary streams are fluvial systems that cease to flow over some amount of time or space (Acuña et al., 2014). In many environments, the greatest proportion of temporary streams are located in headwater systems (Dodds et al., 2004), and there is a growing appreciation that temporary headwater streams exert a strong influence on the structure and function of downstream waterbodies (Acuña et al., 2014). For example, temporary systems provide critical habitat, foster unique biota and transfer energy and nutrients (Stehr and Branson, 1938; Williams, 2005; Meyer et al., 2007). Much like other headwater systems, temporary headwater streams link terrestrial landscapes to river networks across space, but also represent a temporal ecotone due to the highly dynamic nature of environmental conditions in these systems (Steward et al., 2012).

Alternating wet-dry states in temporary streams are known to drive environmental gradients and community structure. Moreover, in the transitions between states (i.e., before a temporary stream becomes completely dry or as a stream rewets) environmental conditions are in constant transition. For example, as flow is reduced in a stream and water settles into isolated surface pools, surface water temperature increases, dissolved oxygen decreases, and increased evaporation rates concentrate solutes causing increased conductivity (Smith and Pearson, 1987). This leads to changes in sediments as well, such as decreases in dissolved oxygen and sharper redox gradients with depth and over time. Subsurface sediments (i.e., the hyporheic zone) can maintain elevated moisture content long after surface drying (Schwinning et al., 2011), potentially resulting in hot spots and hot moments of peak biogeochemical activity in wet compared to dry sediments (McClain et al., 2003). Residual moisture left in the hyporheic zone has been known to help sustain habitat refugia for macroinvertebrates taxa, which rely on moist conditions (Stubbington, 2012; Williams and Hynes, 1974).

The ecological impact of drought as a disturbance in temporary streams has been previously explored (Lake, 2003) as have the responses of fish (Labbe and Fausch, 2000; Dodds et al., 2004; Wigington et al., 2006; Colvin et al., 2009) and macroinvertebrate communities (Boulton and Lake, 1992; Stanley et al., 1994; Fritz and Dodds, 2004; Collins et al., 2007). The variable and often unpredictable hydrologic regime in temporary systems may be a driving force behind many ecosystem processes mediated solely by microbial communities. In such a transitional environment, microbes represent a continuum from truly terrestrial communities in soils to aquatically adapted taxa in streambed sediments. However, unlike other biotic components of temporary systems, generalizable relationships between microbial community structure and environmental gradients have not been firmly established. The lack of relationships are likely complicated because microbial community shifts have also been associated with other environmental factors such as organic matter quality (i.e., leaf litter composition; Artigas et al., 2011; Bruder et al., 2011), conductivity (Zeglin et al., 2011) and sediment composition in addition to the degree of desiccation (Marxsen et al., 2010). Thus, local environmental conditions can interact with temporary stream drying resulting in varying responses across ecosystems.

Shifts in stream conditions—such as a drought and rewetting—serve as filters on community structure. Microbial communities can exhibit resilient, resistant or functionally redundant responses (*sensu* Allison and Martiny, 2008) and thereby affect ecosystem processes. For microbes in temporary streams, resistance to and resilience from drought differ in that the dispersal mechanism is passive and facilitated by water flow. Although it has been well-established that the majority of microbial cells among streambed sediments are destroyed by drying events (Van Gestel et al., 1992), drying can take time and the effect of drying as a filter on microbial community structure is less clear. Long periods of desiccation may induce significant responses by microbial communities than brief events. For resistant communities, disturbance from drought causes little or no change to microbial community composition, whereas resilient communities are impacted by disturbance but are quickly restored after disturbance ends (i.e., surface water is restored). A rapid restoration of microbial processing after substantial portions of the community are lost during a drying event implies that resilience is an important trait in these highly dynamic temporary stream environments.

Equally, rewetting of a temporary stream environment can serve as an environmental filter on microbial community structure. Following rewetting of sediment and soils, microbial processing rates are higher than equivalent sediments not subjected to drying (Soulides and Allison, 1961; Van Gestel et al., 1992). This rapid processing may be driven by microbial communities accessing resources from cells that were destroyed during drying (Van Gestel et al., 1992). For heterotrophs, community composition can be driven by resources that become released or altered upon rewetting, such as the nature of organic matter released (Judd et al., 2006). Taxa that are adapted to rapidly access any available resources may be favored and may maintain a competitive advantage even after stream flow is fully restored.

Research to date characterizing microbial communities on either side of the wet-dry transition has yielded conflicting results. Some studies suggest that microbial community structure showed little difference before and after drying (Amalfitano et al., 2008; Zoppini et al., 2010). Other studies observed substantial depletion of microbial diversity (Timoner et al., 2014b) and substantially altered community composition (Rees et al., 2006; Timoner et al., 2014b) after drying. Similarly, several studies found microbial communities of temporary streams to be resilient, quickly regaining functional activity upon re-saturation of sediments (McIntyre et al., 2009; Timoner et al., 2014a,c). By contrast, other research observed depleted microbial activity for extended periods following flow restoration (Rees et al., 2006). These studies have been typically conducted in a single system or systems within the same region. Moreover, the existing studies suggest that flow cessation of a temporary stream does not necessarily result in a discrete state change to microbial communities but perhaps a more continuous shift in community structure. The lack of a definitive microbial response to wet-dry dynamics in temporary streams suggests that other environmental factors play an important role in these systems, and a regional to global comparison of systems may be warranted.

In practice, the ability to rigorously test hypotheses in temporary streams is a logistical challenge (Lake, 2003). Research efforts are generally hampered by the ability to measure and predict the timing, intensity and frequency of wet-dry events. Unsurprisingly, published datasets are limited in scope and temporal resolution. Moreover human-induced impacts on temporary headwater streams are increasing; true temporary headwater channels are disappearing due to burial (Elmore and Kaushal, 2008) or conversion to perennial status due to urbanization (Roy et al., 2009). At the same time, permanent channels are increasingly becoming temporary and are subjected to more extreme flooding and drying events due to global climate change. This increased variability directly interrupts biotic linkages across the sediment-water interface (Lake et al., 2000). Comparing temporary systems in different regions, using datasets collected from similar environmental conditions, may help address some of these discrepancies and existing knowledge gaps.

We tested the relationships between microbial community structure and environmental conditions, particularly drying and wetting events, in two North American temporary headwater stream systems. Microbial communities were compared for Parkers Creek (Maryland, USA) and Speed River (Ontario, Canada), two different watersheds yet within the temperate zone of eastern North America. Data were collected during different years (Parkers Creek was sampled in 2012; Speed River was sampled in 2007 and 2008). For both systems, we compared sediment and water community composition in streambed sediments before and after seasonal drying events. We further compared stream sediment community composition to that of the stream water column and catchment soils, two sources of colonizing microbes in streams, in order to assess the relative importance of colonization vs. local environmental conditions in sediments.

We predicted that microbial taxonomic diversity in temporary streams would be limited to a subset of groups during periods of stream drying, presumably taxa that are more resistant to desiccation. Thus, we anticipated a substantial shift in community composition and a decrease in taxonomic richness in temporary stream sediments following drying. We also hypothesized that microbial community composition in stream sediments during stream flow would be more similar to water column communities than soil communities. During drying, we expected that the microbial community in stream sediments would change to more closely resemble that of soils. We anticipated that patterns of microbial response to drying would be comparable in both the Speed River and Parkers Creek systems regardless of differing ecosystems and molecular methods. The impacts of temporary stream drying were predicted to persist following rewetting of a stream, a process likely driven by dominance of resistant and resilient taxa that are adapted to dynamic environments.

To place our findings in a broader context, we reviewed published studies and synthesized evidence on the structural and functional response of microbes to drying but found little corroboration across studies due to differences in methodology and analytical resolution among datasets. Therefore, based on the results of this study and our review of the existing literature, three hypotheses are proposed that, when rigorously tested across systems, may strengthen tenuous knowledge of the linkages between environment, community structure, and ecosystem function in temporary headwater streams.

## Methods

### Study sites

#### Speed river site (ontario, canada)

**Figure 1:**
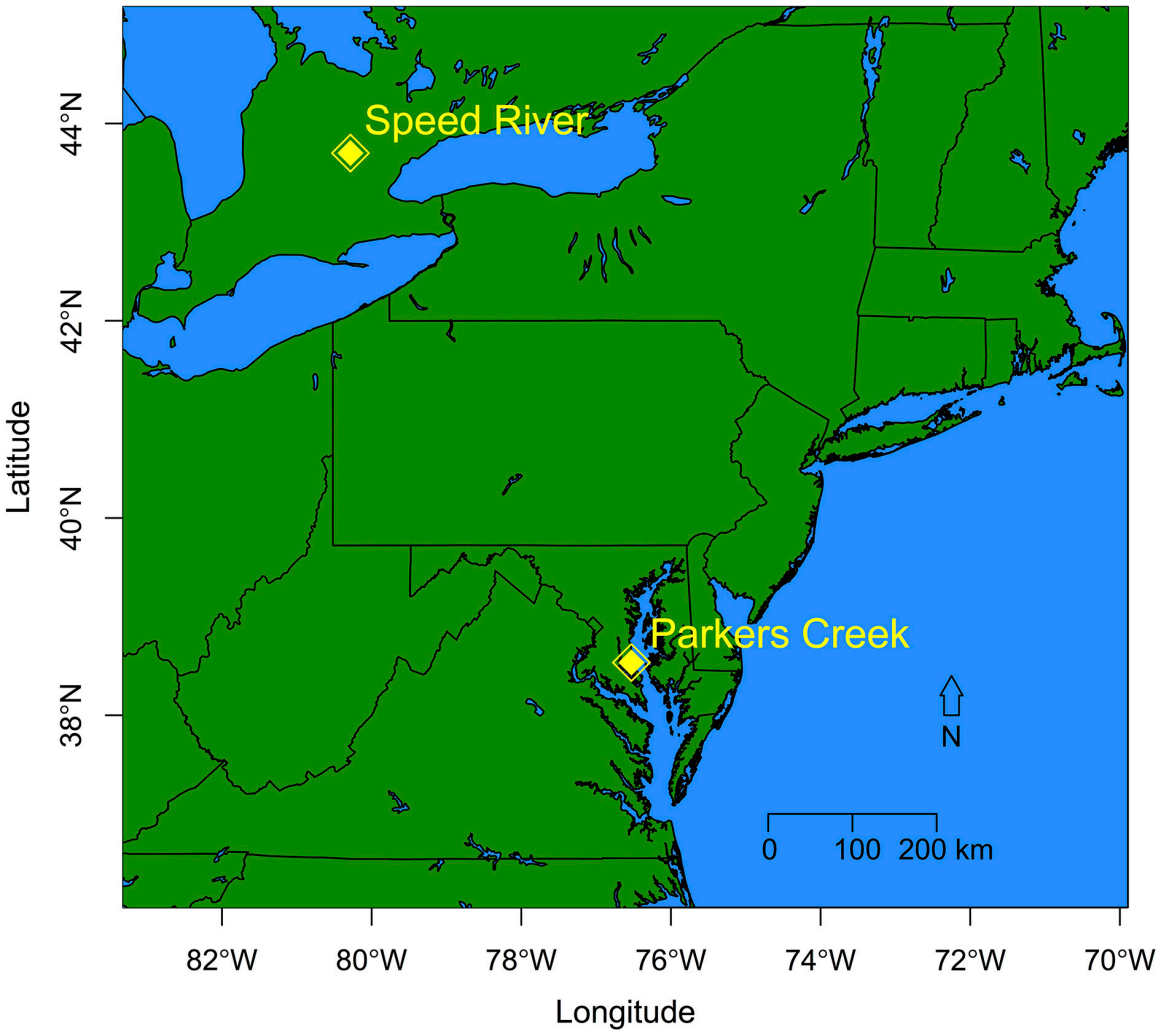
**Location map of the two study systems: Speed River (Ontario, Canada) and Parkers Creek (Maryland, USA)**. See Hosen et al. (2014) and Febria et al. (2010, 2012) for detailed maps of Parkers Creek and Speed River sites, respectively.

The Speed river watershed is a tributary of Lake Ontario. Samples were collected from one permanent (second order) and one temporary (zero order) stream monthly in 2007 and 2008. The stream sites are both tributaries of the Speed River (Permanent site: 43°43′N, 80°16′W; Temporary site: 43°42′N, 80°17′W; Figure 1, Supplemental Table 1). At the permanent site, we focused on a pool-riffle sequence in the stream, measuring approximately 10 m in length and 6 m in width. At the temporary site, we sampled a 15-m section immediately downstream from the springhead that served as the source of the tributary. The temporary stream site was no more than 2 m in width when flooded. Streambed sediments remained saturated throughout the period of study at both sites, however the streambed surface was dry from July to October 2007 and again in July and September 2008.

#### Parkers creek site (maryland, USA)

The Parkers Creek watershed is located in the Coastal Plain in Maryland, USA and drains directly into the Chesapeake Bay (Figure 1, Supplemental Table 1). Samples were collected from three headwater streams sites, two first order stream reaches and one second order stream reach, and are described elsewhere (Hosen et al., 2014). One of the two first order sites was temporary (Site T1: 38°32′51″ N, 76°32′29″ W), the second was permanent (Site P1: 38°33′01″ N, 76°32′39″ W) as was the second order site (Site P2: 38°32′57″ N, 76°32′35″ W).

### Sample collection

At the Speed sites, surface water fluctuated regularly at the temporary site but logging data were not collected due to limited logistical access. Sediment samples were collected monthly in 2007 and 2008 at the permanent site using a standard PVC sampling core (diameter 2.67 cm). Watershed soil samples were grab samples collected on a single occasion (October 2007) from forested and agricultural soils within 100 m of the stream sites (total *n* = 53). At the Parker's sites, both water column and sediment samples were obtained from all sites. Sites P1 and P2 flowed for the duration of the study while surface flow ceased and site T1 became dry from late July through early October 2012 (Supplemental Table 1).

#### Water samples

Stream water temperature, specific conductivity, dissolved oxygen, and pH were determined in the field using a Hydrolab all-in-one Quanta Probe (Hach Inc., Loveland, Colorado, USA) at the Speed site and a YSI Professional Plus multimeter (YSI Inc., Yellow Springs, Ohio, USA) at the Parkers creek site. Water samples from the Speed sites were collected in previously acid washed Nalgene HDPE bottles; water samples from the Parkers Creek sites for chemical analysis were collected in amber borosilicate bottles that had been acid washed and subsequently combusted at 450°C for 4 h and were sealed with Teflon-coated lids. All samples were placed on ice for transport to the laboratory. Samples for genetic analysis were subsequently stored at −80°C prior to further processing. Samples for chemical analysis were stored at 4°C until sample analysis. Dissolved organic carbon and total dissolved nitrogen were determined on stream water samples using a Shimadzu TOC-Vcph total organic carbon analyzer with attached TNM-1 total nitrogen analyzer (Shimadzu Corporation, Tokyo, Japan). The Parkers Creek samples were also analyzed for dissolved organic carbon quality using the fluorescence index (FI), which indicates whether DOM in a water sample is primarily allochthonous or autochthonous (McKnight et al., 2001). The fluorescence index was determined as the ratio of fluorescence emission intensities at 450 and 500 nm when a water sample was excited at 370 nm (McKnight et al., 2001) on a Horiba Scientific Fluoromax-4 (Horiba Limited, Kyoto, Japan). Physicochemical parameters measured at both sites are summarized in Supplemental Table 1.

#### Microbial community composition (speed site)

Sediment samples were stored at −20°C until DNA analysis in the laboratory. DNA was extracted from approximately 1 g of sediment using PowerSoil DNA extraction kits (MoBio Laboratories, Carlsbad, California, USA). Bacterial communities were characterized using terminal-restriction fragment length polymorphism (T-RFLP) and resultant DNA fragments were digested using *MspI* and *HhaI* as described in Febria et al. (2012). Bacterial communities were identified by the different operational taxonomic units (OTUs) and their relative abundance within a given sample. In total, we included only sample dates for which physicochemistry and bacterial community data were available (*n* = 53).

#### Microbial community composition (parkers site)

Water column samples were collected following Crump et al. (2003). Briefly, in the field, 300–600 mL of stream water were passed through a Millipore Sterivex-GP 0.22 μm filter. Residual water was expelled from the filter and approximately 2 mL of DNA extraction buffer were added after which both ports of the filter were sealed. Sediment samples were collected from streambeds to a depth of 3 cm using 2.67 cm diameter sterile plastic coring devices. Twenty cores were taken from random points along a 20 m reach at each site on each sampling date. All cores taken at a site were combined in a single sterile Nasco Whirlpak bag.

Water column microbial DNA was extracted from Sterivex-GP filters using phenol-chloroform based on established protocols (Crump et al., 2003). Filters were defrosted and 20 μL of 1% proteinase-K and 20 μL of 10% lysozyme. Samples were frozen at −80°C for 15 min and then thawed at 37°C for 5 min a total of three times. Samples were then incubated in a water bath for 37°C for 30 min. Fifty μL of 20% filter-sterilized SDS were added to each sample before a 2 h incubation in a 65°C water bath. Samples were washed twice with buffered phenol-chloroform-isoamyl alcohol and then precipitated at room temperature overnight by adding isopropyl alcohol at 60% of sample volume. Microbial sediment DNA was extracted using PowerSoil DNA Isolation Kits (Mo Bio Laboratories, Inc., Carlsbad, CA). To account for the high water content of stream sediment samples, 0.5 grams of sediment was used for each extraction. PCR amplicons were produced using standard methods for high-throughput sequencing (Caporaso et al., 2012). Amplification of 16S rDNA was conducted using forward primer 515f and barcoded reverse primer 806r obtained from the Earth Microbiome Project. For each sample 12 μL of UV-sterilized PCR-grade water, 10 μL 5-prime HotMasterMix, 1 μL 5 mM forward primer, 1 μL of 5 mM reverse primer, and 1 μL of template DNA were combined in a 96-well PCR plate. Conditions for PCR were as follows: Initial denaturation for 3 min at 94°C followed by 30 cycles first at 94°C for 0.75 min, 50°C for 1 min, and 72°C for 1.5 min. At the conclusion of PCR, temperature was held at 72°C for 10 min before temperature was reduced to 10°C. Amplicons were quantified with Pico-Green dsDNA quantification kit (Life Technologies; Carlsbad, CA), combined in equimolar quantities, and cleaned using an UltraClean PCR Clean-Up kit (MO BIO Laboratories, Inc; Carlsbad, CA). Illumina MiSeq 2 × 150 bp sequencing was conducted at Argonne National Laboratory (Lemont, IL).

### Data analysis

#### Both sites

Beta diversity across space and time was analyzed using principal coordinate analysis (PCoA) of Bray-Curtis distances calculated between individual samples. Analysis of similarity (ANOSIM) was conducted to compute similarity between groups. Replication was insufficient to apply statistical tests, but ANOSIM can still be applied to determine the similarity between groups with only one or two members (Cornils et al., 2005). For the Parkers Creek dataset, microbial species richness was estimated using CatchAll (Bunge et al., 2012) on rarified OTU tables. All analysis and plotting were conducted in R version 3.1.2. (R Core Team, 2014). PCoA and ANOSIM were conducted in the vegan package (Oksanen et al., 2013).

#### Parkers site and network analysis

Data were analyzed using the package Quantitative Insights into Microbial Ecology (QIIME). Paired end reads were matched using FLASh (Magoc and Salzberg, 2011). USEARCH 6.1 (Edgar, 2010) was used to identify OTUs at 97% similarity from the Silva 111 database (Quast et al., 2013; Yilmaz et al., 2014) and to identify chimeric sequences. Taxonomy was assigned using the RDP Classifier (Wang et al., 2007) at a threshold of 80%. Sequences were subsequently aligned using PyNAST (Caporaso et al., 2010). Sequences identified as belonging to chloroplasts, mitochondria, and the order Thermales were removed from the dataset as well as any OTU that was not identified taxonomically to at least the class level. Each sample was then rarified to 8960 sequences.

Co-occurrence network analysis of microbial OTU data was applied following existing methods (Barberán et al., 2011; Lupatini et al., 2014; Widder et al., 2014; Williams et al., 2014). To avoid spurious correlations and to aid in the interpretation of results, low abundance taxa that represented less than 0.1% of total sequences were filtered prior to analysis. Pairwise correlations were calculated for each pair of OTUs using Spearman's rank correlation. For a co-occurrence event to be included in the final network a threshold of ρ > 0.75 and *p* < 0.05 was adopted. To confirm that the network generated is not the product of random correlations, a comparison was made with randomly generated networks following (Lupatini et al., 2014). One thousand random networks with size (i.e., number of nodes and vertices) equal to the network generated from the microbial dataset were produced using the Erdös-Rényi model (Erdős and Rényi, 1960). Mean clustering coefficient, mean path length, and network modularity were calculated for each randomly generated network and were compared to the values generated from the experimentally derived network. The *p*-value of rejecting the null hypothesis that the experimental network was obtained at random was calculated as the proportion of values derived from the randomly generated models that were greater than the values obtained from the experiment for each of the three test statistics. For all three statistics the *p*-value was less than 0.001, indicating the experimental network was not obtained at random.

For each node network, centrality metrics including degree, closeness centrality, and betweenness centrality were calculated. Degree is defined as the number of vertices connected to a node. Betweenness centrality is defined as the number of geodesics that pass through a node when all possible geodesics are considered. Closeness centrality is calculated as the inverse of the average length of all the geodesics connecting one node to each other node in the network (Freeman, 1978). These metrics have the potential to identify keystone species within community networks (Williams et al., 2014). Microbial co-occurrence network analysis on simulated communities with known relationships indicate that both node degree and closeness centrality are positively linked to keystone taxa (Berry and Widder, 2014). Network analysis was conducted using R 3.1.2 with the *vegan* and *igraph* (Csardi and Nepusz, 2006) packages.

## Results

### Speed site (ontario, canada)

Monthly samples over a two-year period confirmed differences in several environmental parameters between the permanent and temporary stream tributaries of the Speed River. Several parameters were found to be higher at the temporary than the permanent stream: conductivity (temporary stream = 542 μS/cm, permanent stream 431 μS/cm), total dissolved nitrogen concentrations (temporary stream = 4.63 mg L^−1^, permanent stream = 1.86 mg L^−1^), and dissolved organic carbon concentrations (temporary stream = 8.6 mg L^−1^, permanent stream = 4.6 mg L^−1^; Supplementary Figure 1).

**Figure 2:**
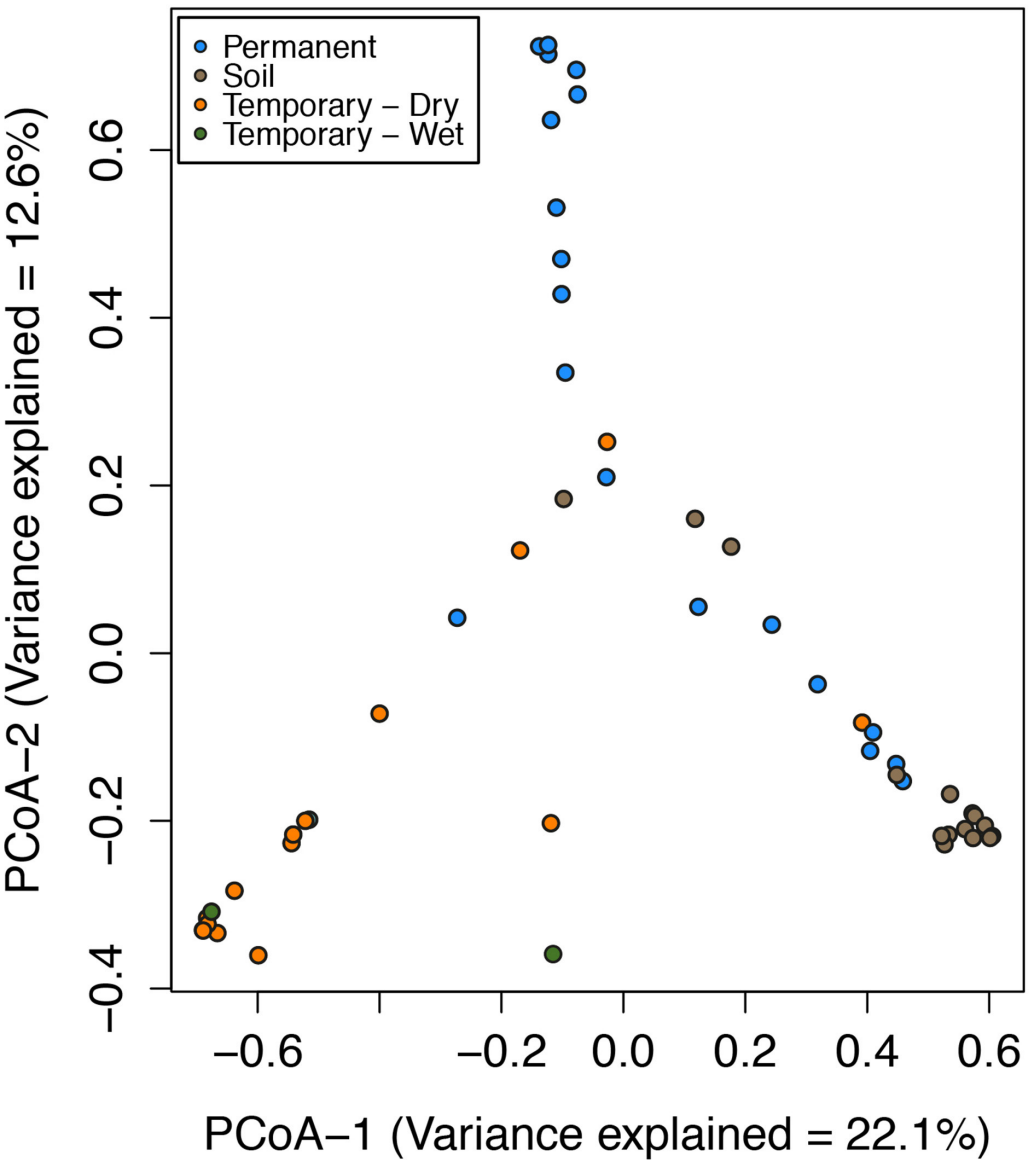
**Ordination of the first two axes of the principal coordinate analysis (PCoA) of microbial community composition Bray-Curtis distances for the Speed River sites**. Indicated are communities from multiple dates at the permanent stream, temporary stream, and watershed soils. Points closer together in the plots indicate similar community composition than that those farther apart. Variance explained in PCoA Axis 1 = 22.1% and PCoA Axis 2 = 12.6%.

In the Speed River system, temporary stream sediment microbial communities were highly similar despite seasonal and hydrologic variation over a 2-year period. The most noticeable difference was that despite the environmental changes, sediment microbial communities were most related to specific sites and sample types (i.e., permanent, temporary, or soil; Figure 2) and not necessarily season or hydrological status. PCoA revealed that sediment and soil microbial community composition were in large part distinct among sites. Further, wetting and drying did not appear to impact community composition of temporary stream sediments. Samples from both wet and dry sediments had similar PCoA scores.

**Figure 3:**
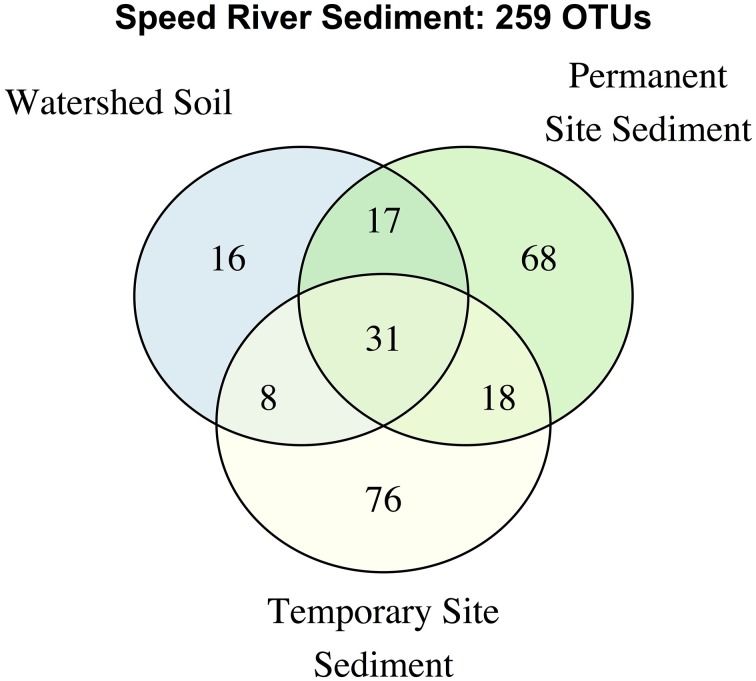
**Venn diagram describing overlap in OTUs in the complete dataset**.

We further examined the distribution of taxa shared across stream types and soils (Figure 3). The number of unique OTUs was roughly comparable in the permanent and temporary stream (134 and 133, respectively) and greater than in watershed soil (72 OTUs). Thirty-one OTUs were shared among the three habitat types with a large proportion of the total number of OTUs found in samples from at least two sites. The permanent stream communities shared a similar number of OTUs with both the temporary and soil habitats (47 and 48, respectively) while fewer OTUs were shared between the soil and temporary stream. Despite being adjacent to riparian areas including watershed soils, the temporary steam sediments had microbial communities that were more similar to the permanent downstream waterway than riparian soils.

### Parkers site (maryland, USA)

Stream water temperatures, DOC and TDN concentrations, and carbon quality (as measured by fluorescence index) were similar across all three sites. At temporary stream site T1, mean conductivity was 316 μS/cm, which was substantially higher than sites P1 (137 μS/cm) and P2 (139 μS/cm). Dissolved oxygen levels were consistently lower at site T1 (mean: 6.0 mg/L) compared to sites P1 (8.4 mg/L) and P2 (7.5 mg/L).

**Figure 4:**
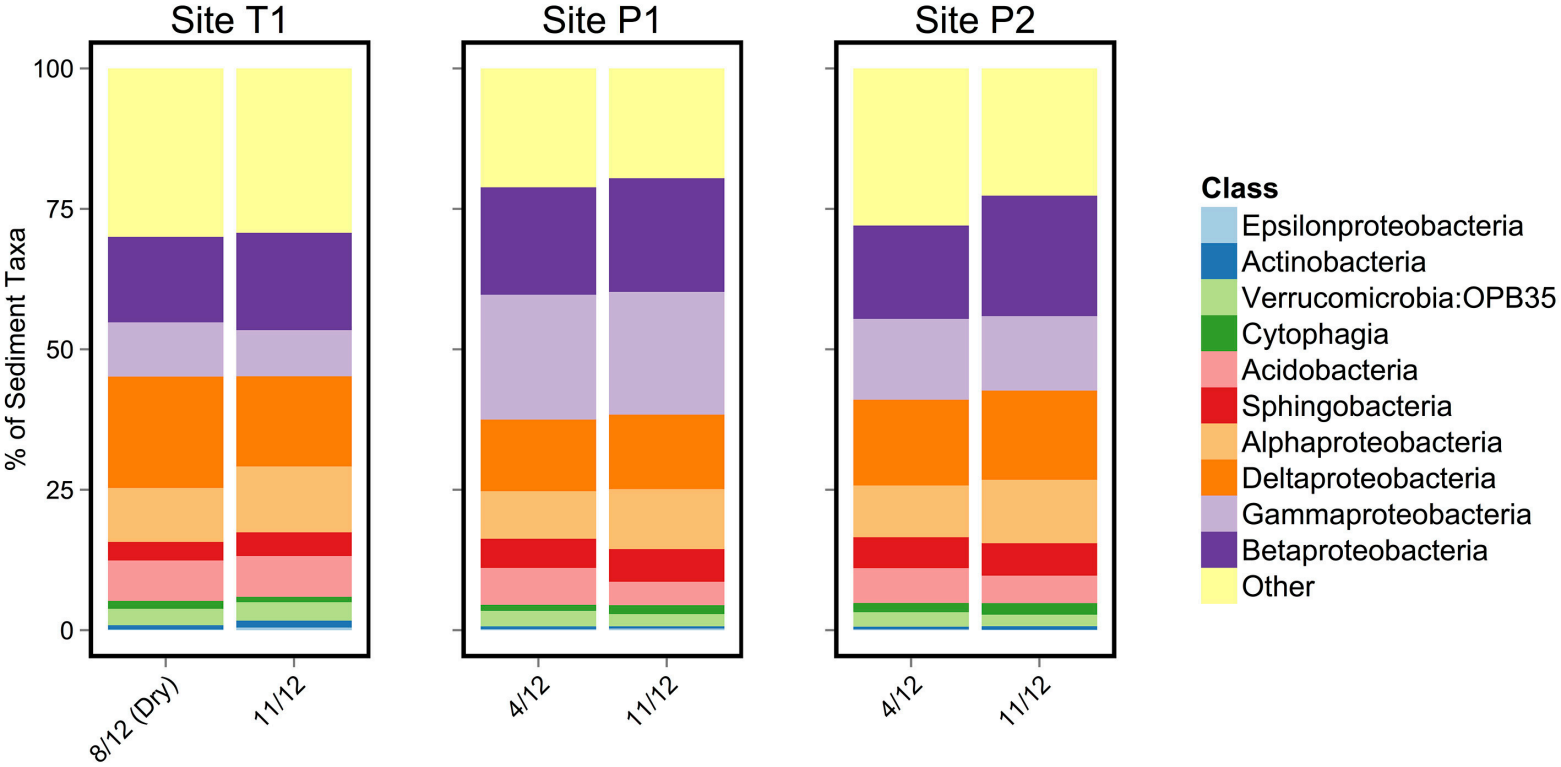
**Class level microbial taxonomic composition of sediment samples**. The 10 most abundant classes are identified in the legend.

**Figure 5:**
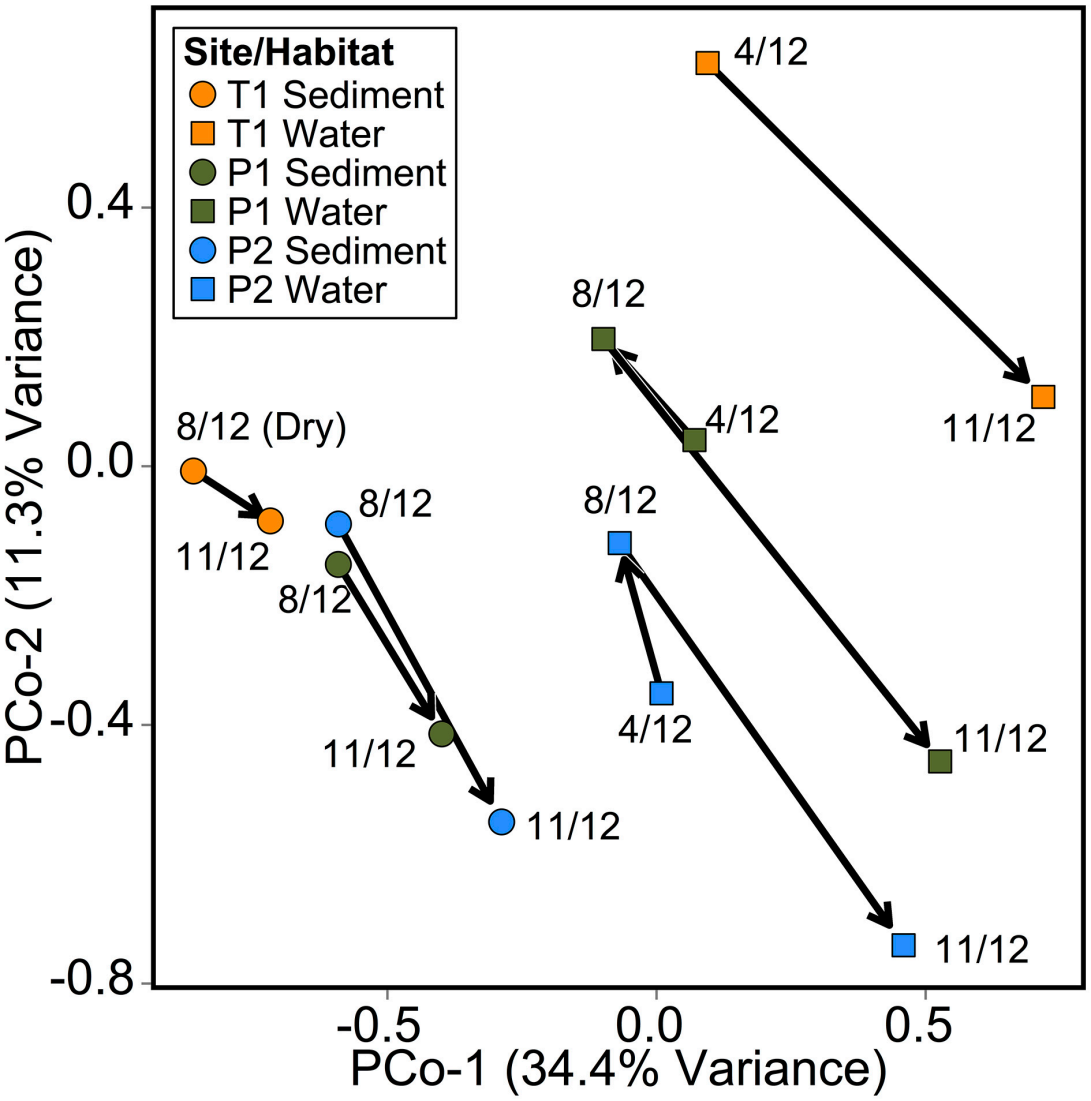
**Principal coordinate analysis (PCoA) of microbial community composition Bray-Curtis distances between samples based on OTU tables**. Points individual microbial sediment or water column samples. Arrows indicate temporal relationship between samples (April 2012, August 2012, and November 2012).

As in the Speed River study, microbial community composition in temporary stream sediments at site T1 did not differ substantially between wet and dry conditions when comparing the percent of sequences belonging to the most common taxonomic classes (Figure 4). To provide a more detailed examination of microbial beta-diversity, PCoA was conducted on the 16S rDNA dataset using Bray-Curtis distances between OTUs for each sample collected and the first two principal coordinates were plotted (Figure 5). The first axis accounted for the 34.4% of variance and separated sediment samples on the left from water column samples on the right. Interestingly, site location appeared to be a stronger factor than stream drying; community composition at site T1 was more similar between the wet (Nov.) and dry (Aug.) seasons than between this site and other sites (Figure 5). This pattern was confirmed by comparing global and pairwise R statistics obtained by ANOSIM. The global R statistic, comparing all four combinations of temporary vs. permanent and sediment vs. water column, was 0.75. Lower R statistic values were identified for sediment vs. water column communities (0.67) and permanent vs. temporary (0.39) comparison.

**Figure 6:**
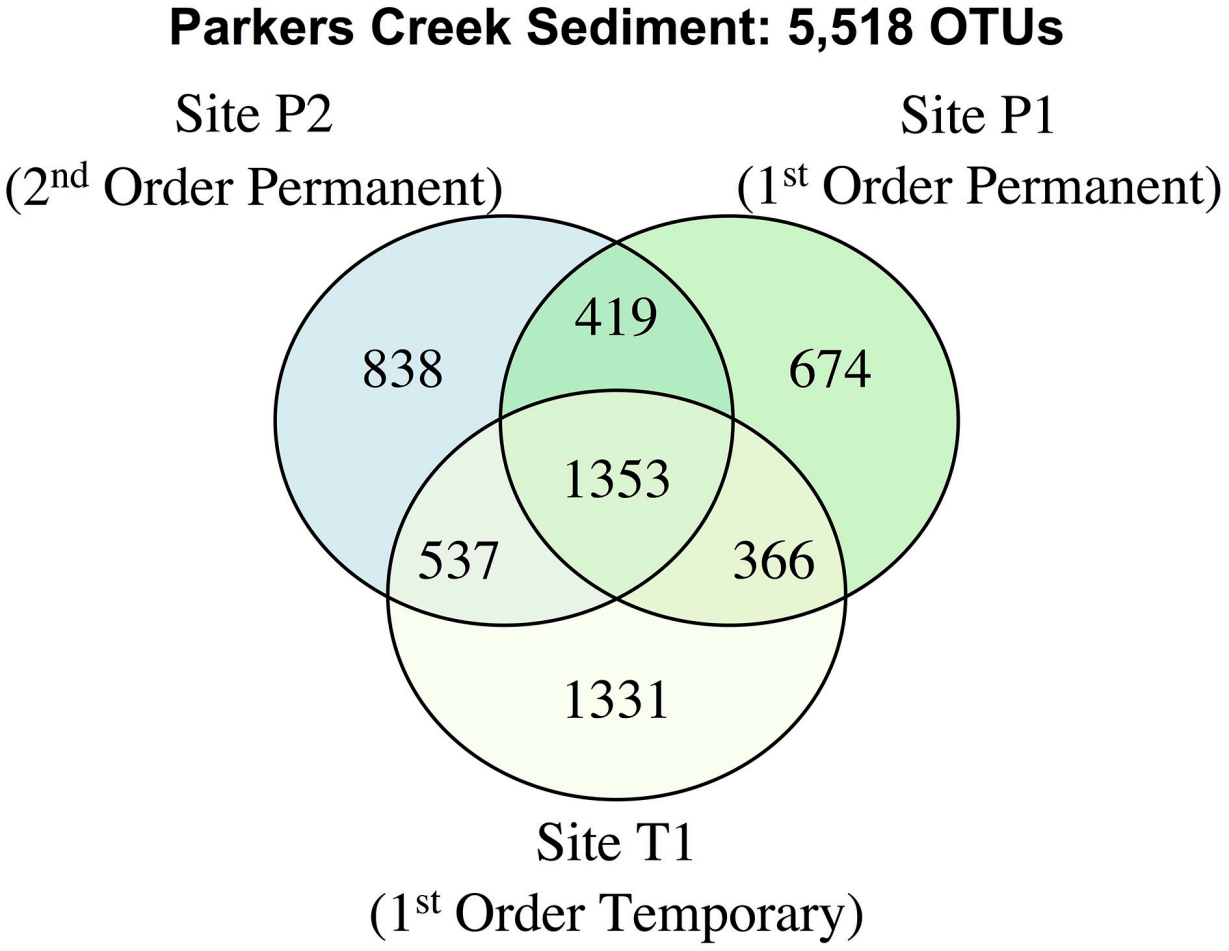
**A venn diagram of sediment OTUs collected from sites T1, P1, and P2 on August and November 2012**.

As in the Speed River system, levels of taxa shared were similar among temporary and permanent sites in the Parkers Creek watershed. Sediment microbial community richness was lower at site T1 during drying conditions in August 2012 (4664; s.e.: 184) than in November 2012 (9523; s.e.: 1693), however this pattern was also true for site P1 which had an estimated 4791 (s.e.: 276) OTUs in August and 6679 (s.e.: 1472) in November. The number of OTUs exclusive to temporary stream site T1 was greater than either permanent stream site (Figure 6). The proportion of shared OTUs in sediments across sampling dates was similar in both first order streams; 30.7% of OTUs at site T1 and 34.8% of OTUs at site P1 were shared between August and November. The microbial community at site P2 appeared to be more stable; 56.7% of OTUs were found in both sediment samples taken at site P2.

#### Network analysis identifies distinct assemblages in permanent and temporary streams

**Figure 7:**
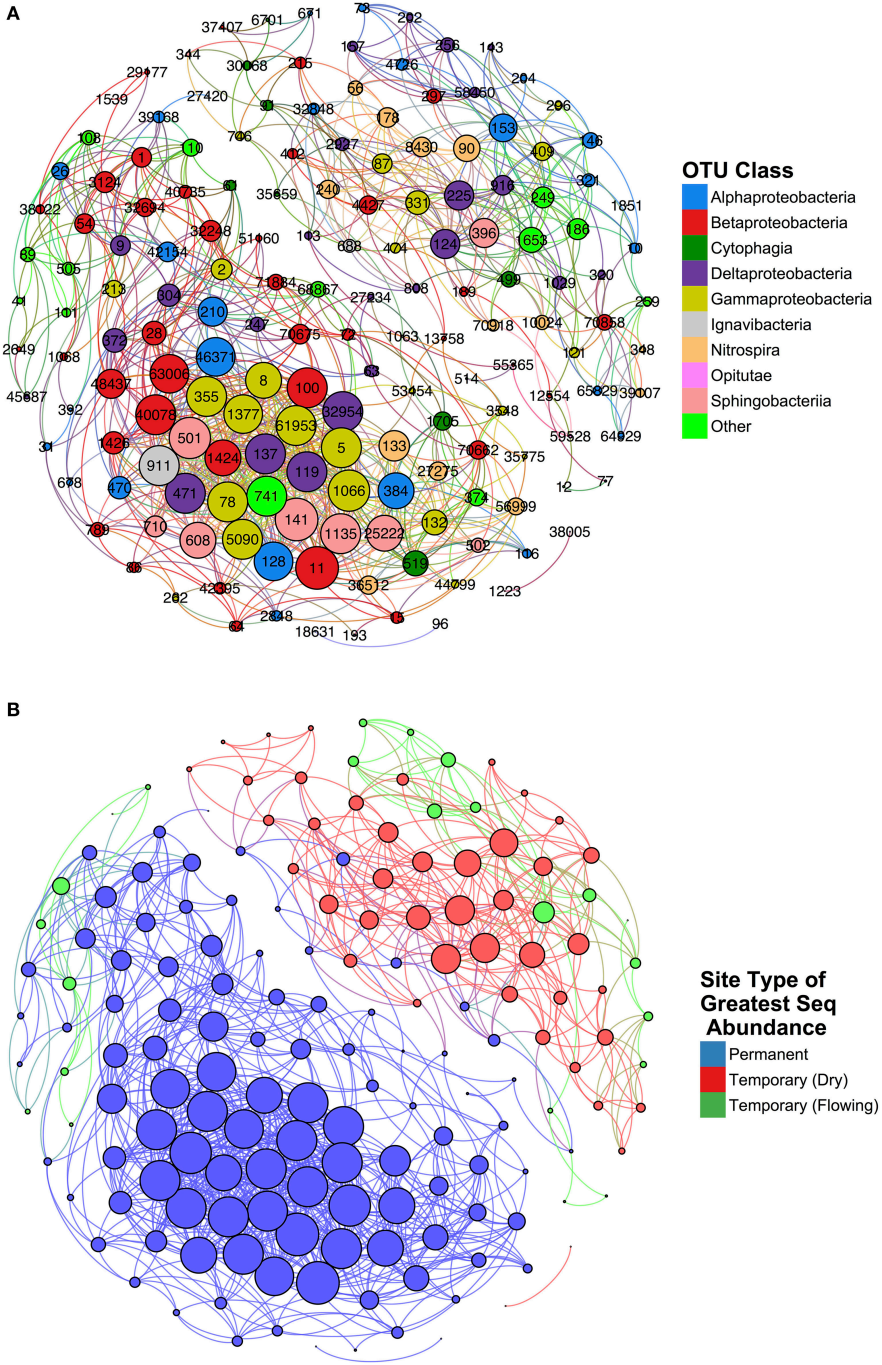
**Results of network analysis conducted on temporary and permanent stream samples**. Nodes represent individual OTUs and edges represent significant spearman correlations (ρ > 0.75 and *p* < 0.05). Node size is determined by weight of that node (i.e., the number of edges connected). Nodes are color-coded according to **(A)** class and **(B)** the site type for which that OTU is most abundant.

Network analysis of microbial co-occurrence patterns incorporated a total of 167 nodes, each representing a distinct OTU, and 1085 edges connecting these nodes. The resulting network clusters OTUs into two groups (Figure 7). Each OTU was labeled according to the site type—permanent, temporary (flowing), and temporary (dry)—and assigned the maximum sequence abundance observed among samples (Figure 7B). The resultant clusters generated represented two distinct communities: one associated with permanent sites, and, a second associated with temporary sites.

The network statistics and taxonomic classification of the OTUs with greatest degree of centrality are identified in Table 1. High-centrality OTUs from the network cluster associated with permanent stream sites were almost entirely members of the Proteobacteria phylum with only one of the top ten OTUs from this group belonging to Bacteroidetes. Seven of the ten permanent stream OTUs were from the order Methylococcales, which is comprised of methantrophs (Bowman, 2005). Another permanent stream OTU that had high network centrality was from the order Methylophilales, a group of methylotrophic bacteria (Qiu et al., 2009). The OTU with the highest closeness centrality was from the family Nitrosomonadaceae; a family that exclusively includes ammonia oxidizing bacteria (Arp et al., 2007).

**Table 1:** **Parkers Creek PCoA loadings of principal coordinate axis 1 and principal coordinate axis 2 for the taxa with greatest loadings from PCoA displayed in Figure 4**.

**Taxonomy**	**PCo-1**	**PCo-2**	**Description of Taxa**
Bacteria; Proteobacteria; Betaproteobacteria; Burkholderiales; Comamonadaceae	2.2383	−1.4288	Family *Comamonadaceae* have been identified as primary denitrifiers in activated sludge (Khan et al., 2002) and associated with younger soils following deglaciation (Nemergut et al., 2007). Multiple taxa in this family have been identified as motile rods (Willems et al., 1991; Khan et al., 2002; Spring et al., 2005). Also includes iron reducing bacteria (Ramana and Sasikala, 2009).
Bacteria;Proteobacteria; Betaproteobacteria; Nitrosomonadales; Gallionellaceae	1.1895	0.7054	Iron-oxidizing bacteria commonly found in streams (Hedrich et al., 2011; Reis et al., 2014).
Bacteria; Proteobacteria; Betaproteobacteria; Burkholderiales; Burkholderiaceae	0.8038	−0.6179	Found with increasing depth in soils (Sait et al., 2002). Includes pathogenic taxa (Ulrich et al., 2006).
Bacteria; Actinobacteria; Actinobacteria; Frankiales; Sporichthyaceae	0.7306	−0.4518	Slow growing taxa associated with compost (Normand, 2006). Grows on a medium containing soil humic acid as the sole source of carbon (Suzuki et al., 1999).
Bacteria; Proteobacteria; Gammaproteobacteria; Xanthomonadales; Sinobacteraceae	−0.7111	−0.0170	Includes non-motile gram-negative taxa obtained from polluted soils (Zhou et al., 2008).
Bacteria; Proteobacteria; Epsilonproteobacteria; Campylobacterales; Helicobacteraceae	0.4602	0.5021	Includes potential anaerobic, nitrate-reducing taxa found in the Baltic Sea (Labrenz et al., 2007) and wetlands in rural Spain (Ansola et al., 2014).
Bacteria; Proteobacteria; Gammaproteobacteria; Methylococcales; CABC2E06	0.6389	0.1747	Includes type 1 methanotroph taxa that have been found in association with iron-oxidizing bacterial communities in riparian wetlands (Wang et al., 2012) and anoxic reservoir water (Quaiser et al., 2014).

**Figure 8:**
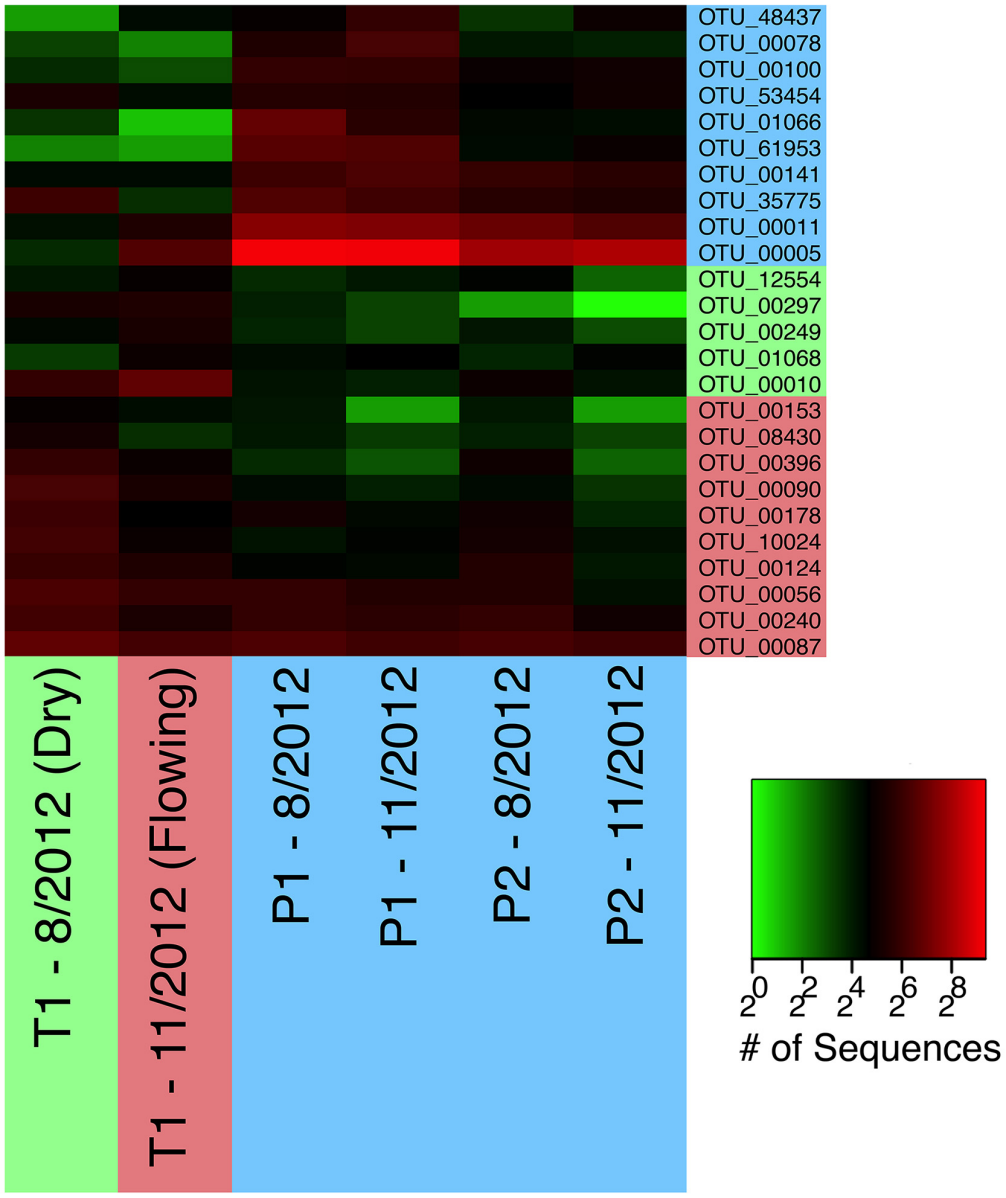
**A heatmap of OTUs presented in Table 2**. OTUs are color-coded as in Figure 7B to indicate whether they are most common in permanent (blue), wet temporary (green), or dry temporary (red) samples. Samples are also color coded using the same scheme.

In contrast to the network cluster associated with permanent stream sites, the temporary stream cluster included a much lower proportion of Proteobacteria OTUs. Six out of the 10 most central OTUs were from the Phylum Nitrospirae, which is dominated by nitrite-oxidizing bacteria often found in wastewater treatment systems (Daims et al., 2001). A heatmap of the OTUs presented in Table 2 (Figure 8) shows that following re-wetting at site T1 (11/2012), OTUs associated with the dry temporary stream decreased in sequence abundance. Despite this shift there were no concomitant increases in sequence abundances of OTUs associated with permanent stream samples.

**Table 2:** **The network OTUs identified with greatest centrality as defined by closeness, centrality, and node degree**.

**OTU ID**	**Closeness Centrality**	**Betweenness Centrality**	**Degree**	**Site type of greatest OTU sequence abundance**	**Taxonomy**
OTU_48437	0.12	937	22	Perennial	Proteobacteria|Betaproteobacteria|Burkholderiales|Comamonadaceae
OTU_00078	0.127	67.4	30	Perennial	Proteobacteria|Gammaproteobacteria|Methylococcales|Crenotrichaceae|Crenothrix
OTU_00100	0.132	811.8	30	Perennial	Proteobacteria|Betaproteobacteria|Nitrosomonadales|Nitrosomonadaceae|Unclassified
OTU_53454	0.129	2385.5	5	Perennial	Proteobacteria|Gammaproteobacteria|Methylococcales|Crenotrichaceae|Crenothrix
OTU_01066	0.132	820	31	Perennial	Proteobacteria|Gammaproteobacteria|Methylococcales|Methylococcaceae|Methylosoma
OTU_61953	0.132	811.8	30	Perennial	Proteobacteria|Gammaproteobacteria|Methylococcales|Crenotrichaceae|Crenothrix
OTU_00141	0.127	106.9	32	Perennial	Bacteroidetes|Sphingobacteriia|Sphingobacteriales|Chitinophagaceae|Terrimonas
OTU_35775	0.129	1029.8	4	Perennial	Proteobacteria|Gammaproteobacteria|Methylococcales|Crenotrichaceae|Crenothrix
OTU_00011	0.127	221.2	32	Perennial	Proteobacteria|Betaproteobacteria|Methylophilales|Methylophilaceae
OTU_00005	0.128	175.5	30	Perennial	Proteobacteria|Gammaproteobacteria|Methylococcales|Crenotrichaceae|Crenothrix
OTU_12554	0.103	320	4	Temporary (flowing)	Proteobacteria|Betaproteobacteria|Rhodocyclales|Rhodocyclaceae
OTU_00297	0.11	131.4	11	Temporary (flowing)	Proteobacteria|Betaproteobacteria|Nitrosomonadales|Gallionellaceae
OTU_00249	0.108	281	16	Temporary (flowing)	Spirochaetes|Spirochaetes|Spirochaetales|Spirochaetaceae|Spirochaeta
OTU_01068	0.104	34.1	6	Temporary (flowing)	Proteobacteria|Betaproteobacteria|Burkholderiales|Comamonadaceae
OTU_00010	0.105	237.2	8	Temporary (flowing)	Proteobacteria|Alphaproteobacteria|Rhizobiales|Bradyrhizobiaceae
OTU_00153	0.113	581.3	21	Temporary (dry)	Proteobacteria|Alphaproteobacteria|Rhizobiales|Xanthobacteraceae|Unclassified
OTU_08430	0.12	494.2	15	Temporary (dry)	Nitrospirae|Nitrospira|Nitrospirales|4-29|Unclassified
OTU_00396	0.117	450.7	22	Temporary (dry)	Bacteroidetes|Sphingobacteriia|Sphingobacteriales|Saprospiraceae|Unclassified
OTU_00090	0.113	256.6	20	Temporary (dry)	Nitrospirae|Nitrospira|Nitrospirales|Nitrospiraceae|Unclassified
OTU_00178	0.12	494.2	15	Temporary (dry)	Nitrospirae|Nitrospira|Nitrospirales|Nitrospiraceae|Unclassified
OTU_10024	0.111	469.7	11	Temporary (dry)	Nitrospirae|Nitrospira|Nitrospirales|4-29|Unclassified
OTU_00124	0.117	450.7	22	Temporary (dry)	Proteobacteria|Deltaproteobacteria|Syntrophobacterales|Syntrophaceae|Syntrophus
OTU_00056	0.118	822.4	11	Temporary (dry)	Nitrospirae|Nitrospira|Nitrospirales|4-29|Unclassified
OTU_00240	0.119	296.6	14	Temporary (dry)	Nitrospirae|Nitrospira|Nitrospirales|Nitrospiraceae|Nitrospira
OTU_00087	0.12	494.2	15	Temporary (dry)	Proteobacteria|Gammaproteobacteria|Xanthomonadales|Sinobacteraceae|Unclassified

*The site type at which the sequences abundance of a given OTU is greatest is also identified*.

## Discussion

### Weak relationship between community composition and drying

The Speed River and Parkers Creek have different climates and exist in landscapes with distinct geologies, yet microbial communities responded similarly to temporary stream drying in both systems. Contrary to our hypothesis, results suggest that drying events did not lead to large changes in microbial communities in streambed sediments. Instead, for both systems, microbial community structure was strongly tied to a location with changes across time apparently related more to seasonal changes than flow status. Thus, the hypothesis that temporary stream sediment communities would resemble soil communities during drying was also not supported as temporary stream communities did not change substantially during drying (Figure 2). In contrast to sediment communities, Febria et al. (2012) found that surface and pore water communities varied significantly within days to weeks of a re-wetting event, whereas sediment microbial communities remained more stable over the same time period and across seasons. Instead community composition was more related to individual sites and site type (i.e., soils, permanent stream sediments, temporary stream sediments).

The weak relationship between community composition and drying was demonstrated in both systems by comparing communities associated with different flow regimes or sample types (e.g., sediments from permanent vs. temporary streams, water vs. sediments). We reported results for comparisons between the four categories (temporary sediment, temporary water, permanent sediment, and permanent water) and confirmed that habitat (sediment and water) or stream type alone (i.e., temporary and permanent) was less compelling. Despite differences between temporary and permanent stream microbial communities at the Parkers site, samples from permanent sites P1 and P2 each shared as many OTUs with temporary site T1 as with each other.

Again at the Parkers site, differences across season were related to seasonal impacts exerted on all samples, rather than a specific effect from stream drying (Figure 4). For example, observed community shifts from August to November was driven by an increase in sequences from the families Comamonadaceae, Burkholderiaceae, Sporichthyaceae, and to a lesser extent Gallionellaceae, and uncultured clone CABC2E06 from the order Methylococcales. There was also a decrease in the number of sequences from the family Sinobacteraceae. These taxonomic shifts from August to November across the study sites suggest establishment of community assemblages associated with iron oxidizing bacteria. Members of the family Gallionellaceae, iron-oxidizers found in stream environments (Hedrich et al., 2011; Reis et al., 2014), increased from summer to fall. The same was true of the family Comamonadaceae, which includes iron-reducing (Ramana and Sasikala, 2009) and denitrifying (Khan et al., 2002) taxa. Another family that increased in sequence abundance from August to November was Helicobacteraceae, which includes potential anaerobic nitrate-reducing taxa that have been found in high abundance in wetland sediments in northern Spain (Labrenz et al., 2007; Ansola et al., 2014). The sequence abundance of Methylococcales clone CABC2E06, a methanotroph that has previously been associated with iron-oxidizing communities also increased from August to November (Wang et al., 2012; Quaiser et al., 2014). Similar assemblages of bacteria have been found in in association with iron-oxidizing bacterial communities in both riparian zones (Wang et al., 2012) and anoxic reservoir water (Quaiser et al., 2014). While more data are needed, this suggests that anoxic conditions near the sediment water interface impact microbial communities in both permanent and temporary headwater streams.

Muted impacts of streambed drying on microbial community structure highlight an important question for future research: What factors control microbial community structure in these streams? In both systems, water column and sediment communities were distinct from sediment microbial communities, suggesting that dispersal via the water column did not have a strong impact on streambed sediment microbial dynamics. While stream drying did not have a strong impact on microbial community composition, environmental gradients associated with drying may play a role. Temporary streams in both the Speed and Parkers Creek systems demonstrated higher stream water conductivity and lower stream water dissolved oxygen compared to permanent reaches. These differences are seen typically in association with drying as evaporation increases solute concentrations and low flows decrease oxygen mixing (Boulton and Lake, 1990; Boulton, 2003). Both conductivity and dissolved oxygen levels have been related to stream microbial community structure in studies of other ecosystems (Lawrence et al., 2004; Zeglin et al., 2011). This suggests that drying may indirectly impact microbial community structure via changes to environmental conditions.

Determining whether functional impact is tied to environmental conditions or the microbial community composition of a temporary headwater stream is an important avenue of future research and beyond the scope of this study and many studies to date. Biogeochemical function may be related more to which fraction of the microbial community is most active, rather than which fraction is the most numerically abundant (Fulthorpe et al., 2008; Shi et al., 2012; Manis et al., 2014). Such a pattern has been observed in studies attempting to link denitrification rates to the abundance of denitrifying taxa. Studies have failed to find a relationship between microbial structure and denitrification rates (Iribar et al., 2008; Song et al., 2012), including one conducted in an ephemeral stream system (Manis et al., 2014). Comparisons between microbial community composition and functional measurements using RNA expression are needed to determine what proportion of temporary stream communities are active and to uncover the sources of functional diversity in temporary streams.

### Central microbial associations differ between temporary and permanent streams

While the overall microbial community structure in Parkers Creek temporary stream sediments was largely similar to the communities in permanent stream sediments, network analysis revealed that different taxonomic associations were dominant in the two types of samples. In permanent stream samples, OTUs from the order Methylococcales and a single OTU from the family Methylophilales were highly central to the microbial network. Taxa from the order Methylophilales do not oxidize methane, but experimental results have indicated that taxa from this group utilizes byproducts of methane oxidation from methanotrophic taxa like Methylococcales (Qiu et al., 2009; Beck et al., 2013; Liu et al., 2014). Similar evidence of cooperative metabolism between the Methylococcaceae and Methylophilaceae families has also been identified (Beck et al., 2013; Liu et al., 2014). Thus, it appears that the network cluster identified in this study represented a community of methanotrophs and other microbes adapted to oxidize methane and associated compounds produced in saturated, anoxic sediments. By contrast, the dominant microbial associations in temporary stream sediments are among OTUs from the order Nitrospirales, indicating that nitrite-oxidizing bacteria play an important role in temporary stream sediments.

Multivariate analysis using Bray-Curtis distances and PCoA demonstrated that bacterial community composition responded weakly or not at all to temporary stream drying. Network analysis presents a useful complement to this approach, revealing that different microbial associations were favored in sediments of temporary vs. permanent streams. Although analysis of overall community composition showed little difference between temporary and permanent stream sediments, network analysis indicated that different assemblages were dominant in the respective stream types and are indicative of functional differences between temporary and permanent stream sediments. Whether this change is representative of functional shifts must be explored with more direct analysis. Network analysis also revealed the presence of microbial associations between taxa that have previously been identified experimentally. This result highlights the utility of this approach as a screening technique to identify previously unrecognized microbial associations.

### Published studies of temporary stream microbial communities yield conflicting results

The results reported here indicated that temporary stream drying and rewetting is not strongly related to shifts in sediment microbial communities of Speed and Parkers sites. To place these findings in context, we compared the results presented here with the limited but growing dataset from temporary headwater streams across the globe (Table 3). This review found that temporary stream studies, including those reported here, report a range of responses to changes in temporary stream flow status.

**Table 3:** **A summary of microbial community compositional and functional studies on temporary streams and general relationships established**.

**Citation**	**Study system**	**Microbial community metric(s)**	**Microbial functional metric(s)**	**Primers or probes used**	**Summary of results**
Amalfitano et al., 2008	Mulargia River (Sardinia, Italy), River Krathis (Peloponnesus, Greece), River Pariela (Portugal), River Tagliamento (Italy).	Bacterial abundance and biomass: DAPI; Community composition: Fluorescence in-situ hybridization (FISH), Automated ribosomal intergenic spacer analysis (ARISA).	Bacterial production ([3-H]leucine incorporation).	Probes: ARCH915, EUB338, EUB338-II, ALF1b, BET42a, GASM42a, PLA46a, CF319a, HGC69a, LGC354abc.	Following experimental desiccation, bacterial carbon production and biomass decreased strongly. Limited change in community structure with increase in Alphaproteobacteria and Betaproteobacteria with drying.
Frossard et al., 2012	Terrestrial soils, ephemeral and permanent stream channel sites in the Chicken Creek watershed (Germany).	Community composition: Denaturing gradient gel electrophoresis (DGGE).	Extracellular enzyme activity: Phosphatase, β-glucosidase, β-xylosidase, cellobiohydrolase, chitinase, leucine-aminopeptidase, aspartate-aminopeptidase, glutamate-aminopeptidase, phenol oxidase, phenol perioxidase.	Primers: Eub338f/Eub518r	Bacterial community structure did not show differences between permanent and ephemeral stream sediments. Enzyme activity was seasonally variable but was not related to microbial community composition.
Febria et al., 2012	In-stream colonization corers at 1 permanent and 1 temporary headwater stream (Ontario, Canada).	Community composition: 16S T-RFLP.		Primers: 27F/1492R.	Strong temporal differences in hyporheic porewater community structure both before and after a drying event
Fierer et al., 2003	Soils from Sedgwick Ranch Natural Reserve (Santa Ynez, CA, USA).	Community composition: 16S terminal restriction fragment length polymorphism (T-RFLP).		Primers: 8 F hex/1389R.	During experimental drying/wetting cycles community composition was varied by environment. Soils without less history of moisture stress, but not in soils with a history of moisture stress.
Frossard et al., 2013	Succession of microbial community in flumes filled with dry stream bed sediments from the Chicken Creek watershed (Germany).	Community composition: ARISA	Extracellular enzyme activity: Phosphatase, β-glucosidase, β-xylosidase, cellobiohydrolase, chitinase, leucine-aminopeptidase, aspartate-aminopeptidase, glutamate-aminopeptidase, phenol oxidase, phenol perioxidase.	Primers: 1406F-FAM/23Sr.	Strong temporal differences in community structure during succession experiments. Enzyme activity changes were linked to shifts in microbial community structure.
Manis et al., 2014	Survey of known temporary streams in agricultural landscapes (USA).	Community composition: 16S and nosZ T-RFLP and 16S and nosZ quantitative polymerase chain reaction (qPCR). Community abundance: DAPI fluorescence microscopy.	Dentrification enzyme assays.	16S T-RFLP Primers: Eub338F-0-III, Eub338F-I-II/1392R. 16S qPCR Primers: Eub339, Eub339 II/ Eub518. nosZ T-RFLP Primers: nosZ-F-1181/nosZR. nosZ qPCR: nosZ1F/nosZ2R.	Greater denitrification rates were observed in ephemeral vs. perennial channels, but potential denitrification was not correlated to denitrifier abundance.
McIntyre et al., 2009	Barnett Creek (Pilbara region, Western Australia). Ephemeral stream located in lowland floodplain.	Microbial biomass: phospholipid fatty acid (PLFA) analysis.	Carbon mineralization assay; carbon dioxide flux assay; carbon and nitrogen stable isotopes.		Landscape position (e.g., riparian soils, floodplain soils, and channel sediments) was less important to microbial activity than soil saturation once water content was greater than 40%. Mineralization of carbon and nitrogen occurs more slowly following complete saturation of sediments compared to brief events that rapidly stimulate microbial activity.
Rees et al., 2006	Semi-permanent stream near Binalong, New South Wales, Australia	Community composition:16S T-RFLP.		Primers: 27F/1492R.	Community composition varied by hydrological condition and within riffles. Communities were changed after drying and did not recover to pre-drought conditions one month after flow was restored.
Timoner et al., 2014a	Dam Creek (South-East Queensland, Australia). First order intermittent headwater stream.	Bacterial abundance: fluorescence microscopy.	Microbial carbon degradation: BiologEcoPlates.		Before re-wetting biofilms differed based on time since drying. Rewetting rapidly increased biofilm functional diversity and functional patterns became more similar across sites. Low counts of bacteria were found in both wet and dry isolated pools in an intermittent channel.
Timoner et al., 2014b	Fuirosos temporary stream (Spain).	Community composition: 16S pyrosequencing.		Primers: 28F/519R.	Differences between biofilm, shallow streambed hyporheic bacterial communities related to flow, drying stress/desiccation and sediment type.
Timoner et al., 2014c	Fuirosos (Iberian Peninsula, Spain). Third order temporary stream.	Community structure and abundance: Chlorophyll-a concentration, pigment composition.			Chlorophyll-a concentrations went down in response to drying but quickly returned following re-wetting. Tendency toward production of protective carotenoids and desiccation resistance structures (e.g., increased membrane thickness and spore production) during drying.
Zeglin et al., 2011	Onyx River (McMurdo Dry Valleys, Antarctica); Rio Salado (New Mexico, USA). Both ephemeral desert streams.	Community composition: DGGE, Clone library analysis.		Primers: 8F/1391R or 1492R. 519R, 515F, 1100R, and 1492R.	Bacterial diversity at both sites was not correlated with sediment water content but was instead most strongly related to conductivity. Community composition was strongly related to water content.
Zoppini et al., 2010	Mulargia River (Sardinia, Italy). Second-order temporary river.	Bacterial abundance/community composition: FISH, DAPI fluorescence microscopy.	Bacterial production ([3-H]leucine incorporation); extracellular enzyme activity.	Probes: EUB338, EUB338-II, EUB338-III, ALF968, BET42a, GAM42a, CF319a, PLA46a, and LGC354abc.	Metrics including bacterial cell counts, bacterial productivity, and enzyme activity were largely comparable during wet and dry conditions. Community composition was not substantially different between wet and dry conditions. Enzyme activities increased during flooding event.
Febria et al. (This study)	Speed River system, Ontario, Canada.	Community composition: 16S T-RFLP.		Primers: 27F/1492R.	Similar community composition in sediment between sites, highly varied surface water communities.
Febria et al. (This study)	Parkers Creek system, Maryland, USA.	Community composition: 16S Illumina MiSeq.		Primers: 515F/806R.	Community composition similar by site. Seasonal changes in microbial community composition were not linked to flow status.

In a study of two European temporary streams, researchers found that the microbial community structure in sediments was resistant to desiccation and rapidly regained function following re-wetting, whereas sediment communities in a stream another system did not (Marxsen et al., 2010). The authors of that study hypothesized that microbial communities in the first stream were protected by higher sediment moisture content (Marxsen et al., 2010). Sediment composition, size distribution, and organic matter content are just a few of the factors that impact sediment drying rate (Gupta and Larson, 1979). Thus, temporary stream pore water, which has been identified as harboring substantial microbial diversity (Febria et al., 2012), may be an important refuge during brief periods of drying. Given that drying periods are predicted to lengthen with climate change (Brooks, 2009; Palmer et al., 2009), we anticipate that the moisture retention capacity of temporary streambed sediments will determine how individual temporary streams respond to climate change. Antecedent conditions may also explain seemingly contradictory results. Fierer et al. (2003) showed that microbes from soils that had been previously exposed to alternating wet/dry conditions were less impacted by experimental drying and re-wetting.

In the studies reviewed, the methods employed to measure microbial community structure or function varied; community composition was largely measured using techniques based on the amplification of the 16S rDNA gene including clone library analysis (Zeglin et al., 2011), degenerating gradient gel electrophoresis (DGGE; Frossard et al., 2013), terminal restriction fragment length polymorphism (T-RFLP; Fierer et al., 2003; Rees et al., 2006; Febria et al., 2012; Manis et al., 2014; this study, Speed River), automated ribosomal intergenic spacer analysis (ARISA; Amalfitano et al., 2008; Frossard et al., 2013), phospholipid-derived fatty acid (PLFA) analysis (McIntyre et al., 2009), fluorescence in-situ hybridization (FISH; Amalfitano et al., 2008; Zoppini et al., 2010), pyrosequencing (Timoner et al., 2014b), and Illumina MiSeq (this study, Parkers Creek). A wide variety of PCR primers were used, the only primer pair that was repeated across studies was 27F/1492R (Rees et al., 2006; Febria et al., 2012, this study, Speed River; Table 3), which limited our ability for direct comparison across systems. While inconsistent methods may be one factor contributing to conflicting results, it is important to note that the results obtained from these two systems were largely similar despite different community analysis techniques. Thus, at least some of the disparate results found across studies is likely due to true differences in environment.

### Conclusions and future research

In this study, community composition was weakly linked to flow status, with variability in community structure in temporary streams related to other factors. This adds some support to the idea that changes in the function of temporary stream microbes over time is a factor more of changing environmental conditions than shifting microbial community composition. By contrast, network analysis did show that the dominant microbial interactions shifted with stream wetting and drying. This suggests that a subset of the overall microbial community is more responsive to stream flow status than the overall microbial population. Future research should be conducted to determine the functional impacts of these changing associations. Our review of data from the Speed and Parkers systems and others from around the globe suggests that research on sediment microbial communities in temporary headwater streams is a rich but not yet unified pursuit. Identification of key controls on microbial community structure in temporary headwater streams hinders efforts to develop predictive models that elucidate links between microbial structure and function to ecosystem-scale processes and the impacts of human actions on these processes.

Thus, future research should directly address these knowledge gaps by identifying the factors leading to the inconsistent findings highlighted here. We identified the following hypotheses that may explain our results, and when tested across other systems, may fill in critical knowledge gaps and address broader questions about controls on microbial community structure and function in temporary headwater streams and related water management needs:

1. Degree of sediment water retention, not flow status, determines whether a temporary microbial community is resistant to drying. Our analysis suggests that flow status itself had marginal impact on community structure in some temporary headwater streams but substantial impact in other systems. Rather, the degree of sediment drying, which is controlled by a number of factors including sediment composition, may be a more important factor. Prior exposure to highly variable conditions may also play a role. Experimental studies show that soil microbial communities previously exposed to drying change less in response to experimental drying (Fierer et al., 2003). Studies that directly examine these factors are needed to address this issue.
2. Contradictory results can be resolved by standardized field and molecular methods. The collection of physicochemical data including the timing and frequency of wet-dry events is especially challenging. Our limited ability to generalize findings across studies are due to the intermittent nature of surface flow in these headwater systems and the resultant lack of temporal and spatial resolution in the available datasets. For example, in both the Speed and Parkers systems, *in situ* data collection was either logistically infeasible (due to their remote location or unpredictable surface flow conditions), or instrumentation were either damaged or stolen during critical periods. Moreover, the tools with which to characterize microbial communities vary widely, making cross-site comparisons difficult. New technology and lowering costs promise to make high-throughput sequencing a standard practice and allow for more comparable datasets.
3. Functional rates in temporary streams are more related to environmental conditions than to community composition. Evidence reviewed here suggests that microbial community structure is often similar between wet and dry conditions even as the processing rates of some functions, such as denitrification, change between wet and dry conditions. This suggests that environmental changes may alter functional processing rates of stream microbial communities, a pattern that must be tested for other microbial functions.

Despite increasing human-induced impacts on headwater streams, appreciation for temporary streams and the contributions of these systems to ecosystem processes are building. Understanding the critical drivers of microbial community diversity and function in these systems will inform restoration efforts focused on enhancing or supporting nutrient cycling and food web interactions across space and time.

### Conflict of interest statement

The authors declare that the research was conducted in the absence of any commercial or financial relationships that could be construed as a potential conflict of interest.
