# Supplementary material for: Microbial responses to changes in flow status in temporary headwater streams: a cross-system comparison
Source: Front Microbiol. 2015 Jun 4;6:522. doi: 10.3389/fmicb.2015.00522 (PMC4454877; doi:10.3389/fmicb.2015.00522)
Supplement: Supplementary file 2 [file DataSheet1.PDF]

Supplementary Material

Microbial responses to environmental changes in temporary headwater streams: a cross-system comparison and hypotheses for future research

Catherine M. Febria[1,2]*, Jacob D. Hosen[1]*, Byron C. Crump[3], Margaret A. Palmer[1 2,4], D. Dudley Williams[5].

1 = Department of Entomology, University of Maryland, College Park, MD, USA & Chesapeake Biological Laboratory, Solomons, MD, USA
2 = School of Biological Sciences, University of Canterbury, Christchurch, New Zealand
3 = College of Earth, Ocean, and Atmospheric Sciences, Oregon State University, Corvallis, OR, USA
4 = National Socio-Environmental Synthesis Center, Annapolis, MD, USA
5 = Emeritus Professor, University of Toronto Scarborough, 1265 Military Trail, Scarborough ON Canada
* = *Authors declare equal contribution*

*** Correspondence:** Catherine M. Febria, School of Biological Sciences, University of Canterbury, Private Bag 4800, Christchurch 8023, New Zealand. catherine.febria@canterbury.ac.nz.
Jacob Hosen, Chesapeake Biological Laboratory, University of Maryland Center for Environmental Sciences, P.O. Box 38, Solomons, MD 20688, USA. jhosen@umd.edu.

1. Supplementary Figures

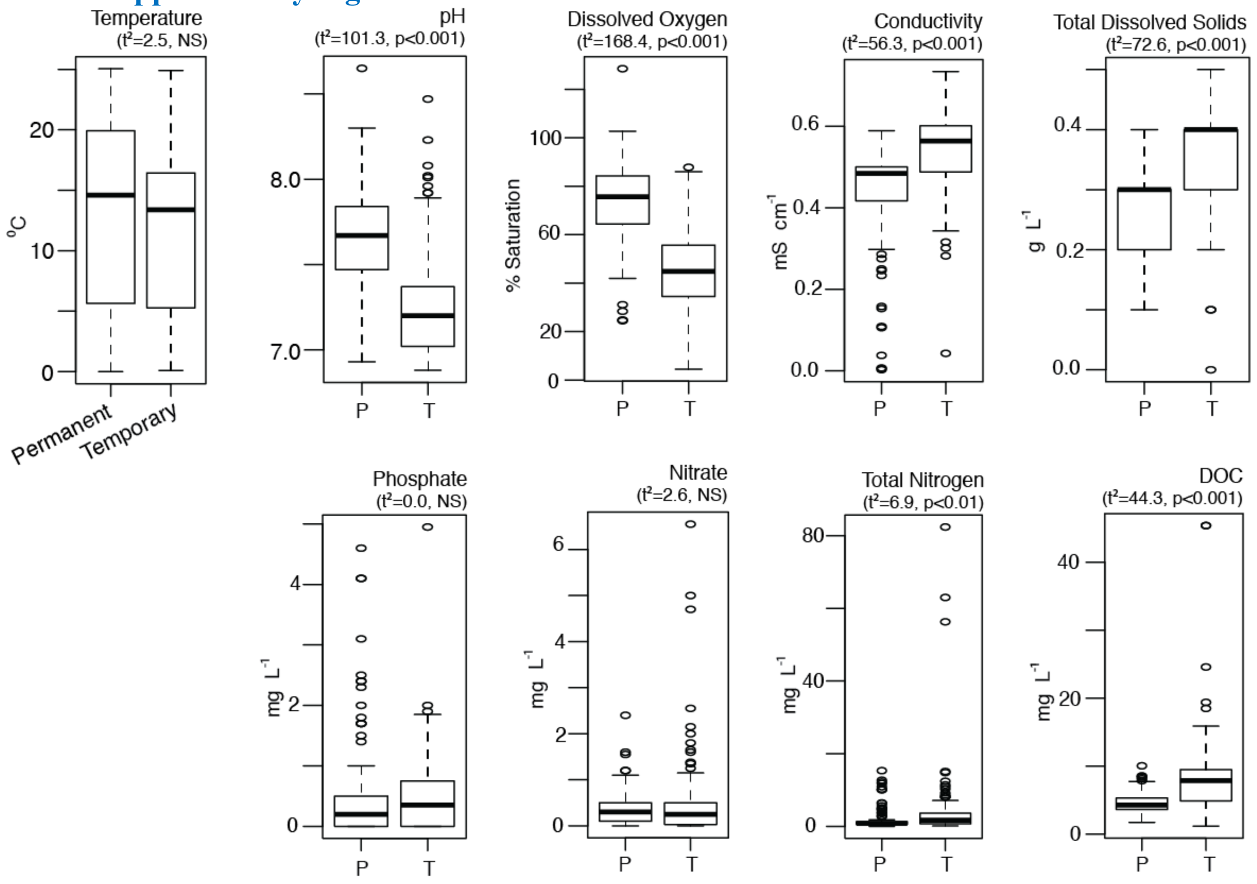

Supplementary Figure 1 – Box whisker plots of monthly physicochemical measurements for the Speed River permanent (P) and temporary (T) stream sites between 2006 and 2008. Water samples include surface water and porewater samples (collected via colonization corers, Febria et al. 2010 & Febria et al. 2012). For each panel, mean annual values (black line), interquartile range (rectangle), whiskers indicating 10th and 90th quartile ranges, and outliers (circles) are shown. T-tests were conducted to test if values were significantly different between streams. t^2 and p-values are provided above each panel.

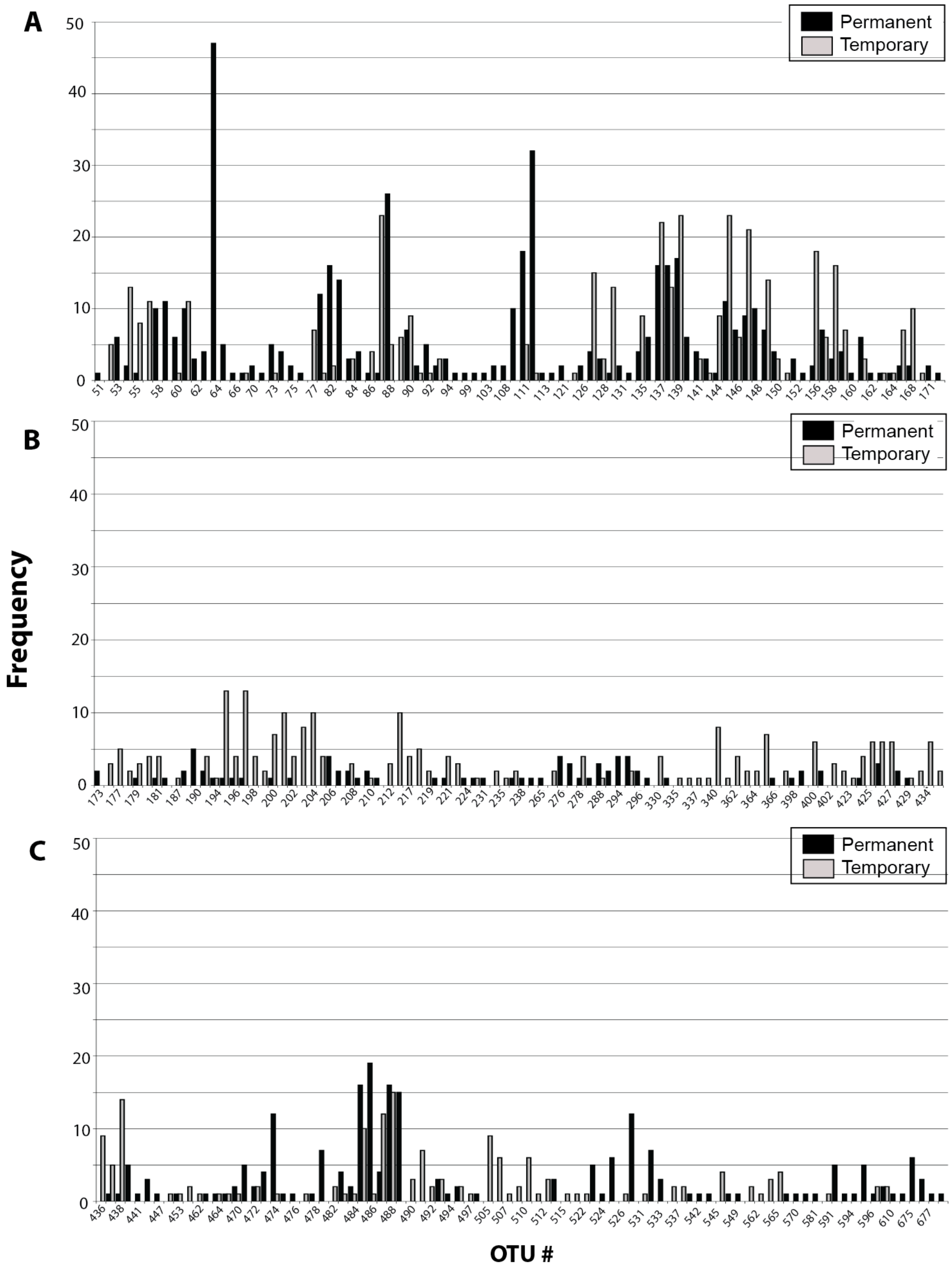

Supplementary Figure 2 - Summary of OTUs described among all samples at the Speed River permanent site (temporaryblack bars) and temporary site (grey bars). Individual OTUs are identified on the x-axis (Panel A: OTU 51 to 172, B: 173 – 435; C: 436 – 829), and the mean frequency of each OTU across samples on the y-axis. OTU number corresponds to the terminal-restriction fragmen position in the electropherogram and only OTU numbers identified in the samples are identified.
